# The Therapeutic Targets of miRNA in Hepatic Cancer Stem Cells

**DOI:** 10.1155/2016/1065230

**Published:** 2016-03-28

**Authors:** Sabrina Bimonte, Maddalena Leongito, Antonio Barbieri, Vitale del Vecchio, Michela Falco, Aldo Giudice, Raffaele Palaia, Vittorio Albino, Raimondo Di Giacomo, Antonella Petrillo, Vincenza Granata, Francesco Izzo

**Affiliations:** ^1^Division of Abdominal Surgical Oncology, Hepatobiliary Unit, National Cancer Institute “G. Pascale Foundation” IRCCS, 80131 Naples, Italy; ^2^Animal Facility Unit, National Cancer Institute of Naples “G. Pascale Foundation” IRCCS, 80131 Naples, Italy; ^3^Epidemiology Unit, National Cancer Institute of Naples “G. Pascale Foundation” IRCCS, 80131 Naples, Italy; ^4^Division of Radiology, National Cancer Institute “G. Pascale Foundation” IRCCS, 80131 Naples, Italy

## Abstract

Hepatocellular carcinoma (HCC) is the fifth most common cancer worldwide malignancy and the third leading cause of cancer death in patients. Several studies demonstrated that hepatic cancer stem cells (HCSCs), also called tumor-initiating cells, are involved in regulation of HCC initiation, tumor progression, metastasis development, and drug resistance. Despite the extensive research, the underlying mechanisms by which HCSCs are regulated remain still unclear. MicroRNAs (miRNAs) are able to regulate a lot of biological processes such as self-renewal and pluripotency of HCSCs, representing a new promising strategy for treatment of HCC chemotherapy-resistant tumors. In this review, we synthesize the latest findings on therapeutic regulation of HCSCs by miRNAs, in order to highlight the perspective of novel miRNA-based anticancer therapies for HCC treatment.

## 1. Introduction

Hepatocellular carcinoma (HCC) is the fifth most common cancer in the world and in most cases it develops in patients with chronic liver diseases such as viral infections and cirrhosis. Treatment for primary liver cancer depends on the location and stage of the cancer and liver's functionality; however, in many cases HCC is diagnosed in patients with an advanced stage. Thus, it is difficult to treat patients at surgical and pharmacological levels [1]. Treatment options include surgical resection, thermal ablation, systemic chemotherapy, transarterial chemoembolization, and selective internal radiation therapy with poor prognosis in all approaches due to the high recurrence rate and to the tumor chemoresistance.

The emerging cancer stem cell (CSC) theory based on targeting of CSCs [2] suggests new alternative therapeutic approaches to treat various types of tumors that could overcome defeats of the traditional therapy. Thanks to the identification of the major signaling pathways, transcriptional factors, surface markers, microRNA (miRNAs), and other factors that confer stem-like properties to CSCs, various therapeutic implications have been developed until now.

Hepatic cancer stem cells (HCSCs) represent a subpopulation of cells positive for different markers including CD133, CD90, and EpCAM [3]. These cells are responsible of the tumor initiation and progression and are also involved in the metastasis processes and chemoresistance. Thanks to the HCSCs characterization, it is possible to study the impact of any molecular mediators highly expressed in HCSCs during carcinogenesis, through the identification of specific stemness markers. The major stem-maintenance pathways involved in HCSCs regulation are TGF-*β* family, Wnt/*β*-catenin axis, PI3K/AKT/mTOR, and EpCAM.

It is of note that these signaling pathways are subjected to different homeostasis system and in particular are regulated epigenetically by miRNAs. MiRNAs, by acting as oncogenes or oncosuppressors, are able to regulate many biological processes such as self-renewal and pluripotency of HCSCs [4, 5], representing a new promising strategy for treatment of chemotherapy-resistant HCC tumors.

In this review, we summarized the latest findings on the therapeutic regulation of HCSCs induced by miRNA and we try to elucidate the underlying mechanisms in order to highlight the perspective of novel miRNA-based anticancer therapies for HCC treatment.

## 2. Hepatic Cancer Stem Cells: Biogenesis and Functions

Cancer stem cells (CSCs) or tumor-initiating cells (T-ICs) are tumor cells discovered in solid and hematological tumors. These cells share stem-like properties and are involved in the tumorigenesis, in development of metastases, and in self-renewal processes. Bonnet and Dick described CSCs for the first time, in acute myeloid leukemia [6]. Several studies showed their existence in other different types of cancer such as glioma [7], breast [8], colon [9], ovarian [10, 11], pancreatic [12], prostate [13, 14], lung [15], liver [16], and stomach [17] cancer.

It has been demonstrated that CSCs play important roles in the tumor initiation and maintenance but also in metastasis and cancer relapse induced by the chemoresistance to the conventional therapies [2]. CSCs can be originated by different processes ranging from the transformation of normal stem cells or progenitor cells through multiple gene mutations [18] or by adult progenitor cells and progressive acquisition of stem cell properties through reversal of ontogeny [19] as the epithelial-to-mesenchymal transition (EMT) process. EMT is a biologic process that induces the transformation of a polarized epithelial cell into a mesenchymal cell type with stem-like properties [20]. In addition, it can also confer to the CSCs the ability to generate metastasis, which is uncommon in other cancer cell types. EMT together with reverse transition from mesenchymal to an epithelial phenotype (MET) are involved in embryonic development, which leads to the disruption of epithelial cell homeostasis and the acquisition of a migratory mesenchymal phenotype [21]. The EMT process is controlled by the canonical pathways such as Wnt and transforming growth factor-*β* (TGF-*β*) [22]. These observations suggest a new concept of migration process based on the existence of two forms of cancer stem cells: (1) stationary cancer stem cells (SCS) and (2) mobile cancer stem cells (MCS). SCSs are involved in tumor initiation and are detectable in the differentiated central area of tumor, while MCSs are known as cells derived from SCSs through the acquisition of EMT.

Hepatic CSCs (HCSCs) have been isolated from heterogeneous tumor tissues, based on the specific surface markers and functional properties. It is of note that various markers have been identified for hepatic cancer stem cells, including CD133, CD90, and EpCAM [3].

The principal HCSCs molecular markers are summarized in Table 1.

## 3. Signaling Pathways Involved in the Regulation of HCSCs

HCSCs show specific features of pluripotency and self-renewal; this phenotype is strictly regulated by different types of molecular effectors involved in many pathways. Here, we review the latest findings on the most important mediators involved in HCSCs regulation.

### 3.1. TGF-*β*

TGF-*β* is a pleiotropic cytokine involved in embryonic development and adult homeostasis maintenance. It regulates the progression of many human diseases as embryo-defects, autoimmune illness, and cancer progression [57–59]. It has been also demonstrated that TGF-*β* regulates HCSCs cell proliferation and differentiation. Its deregulation induces aberrant expression of IL-6 resulting in changed differentiation pattern and tumorigenesis [60]. TGF-*β* binds to the heterodimeric surface receptor T*β*RI/T*β*RII. Subsequently, the subunit T*β*R is activated by the phosphorylation of the C-term motif Ser-X-Ser of R-Smads or Smad2/3. The result is the formation of oligomeric Smad complex (together with Co-Smad and Smad4) that is then accumulated in the nucleus and regulates gene expression [61, 62]. Other studies showed the interaction between TGF-*β* and other cell effectors signal transducer such as MAPKs, ERK, JNK and p38, PI3K/AKT axis, RhoA GTPase, and PAK2 [63, 64]. Interesting data showed that Smad7 highly expressed in hepatocarcinoma and other types of cancer disease [65] negatively regulates TGF-*β* through T*β*RI subunit [66, 67] or by interference with the R-Smad-Smad4-DNA complex formation [68]. TGF-*β* signaling also induces endothelial-to-mesenchymal transition (EMT) in neoplastic cells.

Emerging evidences indicate that Smad7 also regulates Wnt/*β*-catenin, NF-*κ*B, interleukin-1/Toll-like receptor, and EGF/MAPK signaling pathways [65, 66]. Recently it has been demonstrated that HCCs with impaired levels of transcription-3/OCT4 have dysfunctional TGF-*β* signaling and share similar properties of cancer progenitor cells [67].

### 3.2. Wnt/*β*-Catenin

**Figure 1:**
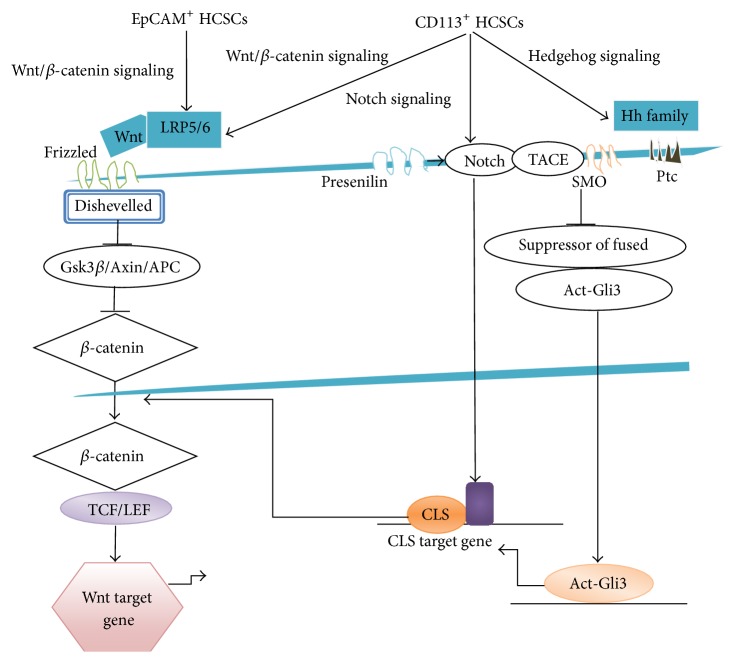
Key signaling pathways that regulate the function of hepatic cancer stem cells. The cartoon recapitulates the principle signaling pathways that regulate the function of hepatic CSCs such as Wnt/*β*-catenin, Hedgehog, and Notch. Epithelial cell adhesion molecule (EpCAM) is a Wnt/*β*-catenin signaling target gene. The activation of Wnt/*β*-catenin signaling regulates EpCAM expression in HCSCs. In CD133^+^ HCC cells, Wnt/*β*-catenin, Notch1, and smoothened (SMO)/Hedgehog signaling pathways are associated with proliferation, self-renewal, and differentiation of HCSCs.

One of the most recurrent pathways involved in HCSCs regulation is the Wnt/*β*-catenin signaling. This signaling regulates development, growth, survival, regeneration, and self-renewal processes in HCC [68]. *β*-catenin acts as a pivotal mediator in Wnt/*β*-catenin signaling pathway through the interaction between Frizzled, the receptor of Wnt, and coreceptor lipoprotein receptor related 5/6 (LRP5/6). This event results in the activation of Disheveled (Dvl), in the dissociation of tetrameric *β*-catenin/Axin/GSK3*β*/APC complex, in the reduction of *β*-catenin phosphorylation, and in the migration of active *β*-catenin to the nucleus (Figure 1).

It has been showed that cytoplastmatic and nuclear accumulation of *β*-catenin were founded in 20–40% of HCC patients, although its target genes were unaffected.

Nuclear *β*-catenin interacts with T-cell factor (TCF)/lymphocyte enhancer factor 1 (LEF1) and some other coactivator as BCL9, Pygo, or CREB-bp to regulate gene transcription of specific sequences [69, 70]. The prominent targets of this signaling are CD44 [71], cyclin D1 [72], and c-myc [73]. c-Myc is considered the preferred target of EpCAM, an adhesion transmembrane glycoprotein, identified as a good marker of HCSCs [74] but also a prognosis biomarker, due to its correlation with more aggressive diseases [75, 76].

These data suggest that Wnt signaling is involved in HCSCs maintenance.

### 3.3. EpCAM

The EpCAM signaling starts with a sequential cleavage of the surface protein, operated by TNF-*α* converting enzyme (TACE/ADAM17) and a gamma-secretase complex containing presenilin-2 (PS-2) (Figure 1). It results in the separation of the extracellular domain, EpEX, and the cytoplasm releasing of EpICD domain that becomes part of multiprotein complex composed of *β*-catenin and LEF (both components present in Wnt/*β*-catenin signaling). A key role in EpCAM pathway is played by FHL2 that first regulates the localization of the cleavage and then acts as a link between EpICD and specific DNA sequences [77]. Some transcriptional factors involved in pluripotent stem cells maintenance, as Nanog, Klf, Sox2, and OCT4, have been described as direct target of EpCAM in human embryonic stem cells [78]. It has been demonstrated that EpCAM is a Wnt-beta-catenin signaling target gene and may be used to facilitate HCC prognosis [79].

### 3.4. PI3K/AKT/mTOR

The PI3K/AKT/mTOR signaling that has been found to be deregulated in 40–50% of HCC cases, with less differentiated tumors and with reduction of free disease survival [80].

Specifically, the activation of IRS1, an intracellular mediator of insulin signaling, induces the activation of PI3K (Phosphatidylinositol 3-Kinase). This leads to the phosphorylation of PKB (protein kinase B)/AKT mediated by PDK1 (Pyruvate Dehydrogenase Kinase Isozyme 1), a positive regulator of the tuberous sclerosis (TSC1-TSC2) complex; the latter promotes the activation of mTORC1, a mammalian target of rapamycin complex 1, through the small GTPase Rheb (Ras homolog enriched in brain). mTORC1 can target and activate S6K1 (ribosomal protein S6 kinase) and 4E-BP1 (eukaryotic initiation factor 4E binding protein 1), major regulators of protein translation. The Phosphatase PTEN (Phosphatase and tensin homolog) physiologically inhibits the downstream activity of PI3K/AKT axis and is frequently deregulated in HCC (66% of tumor incidence in PTEN-deficient mice) [81]; moreover, it is correlated with poor prognosis and more frequent metastasis [82].

### 3.5. Hedgehog

Hedgehog pathway plays an important role during embryonic development and in cell fate maintenance. It is activated by binding of ligands (Desert, Indian, and Sonic Hedgehog) to the membrane based patched (Ptc) receptors [83, 84] (Figure 1). Recent reports established the role of Hedgehog signaling in HCC [85–87].

Figure 1 shows a summarized view of pathways involved in regulation of HCSCs.

## 4. Therapeutic Targets of miRNA in Hepatic Cancer Stem Cells

Recent studies showed the role of miRNA in many biological processes, including the regulation of carcinogenesis, sharing both oncogenes and oncosuppressor functions [36, 54, 88–90]. The deregulation of miRNA expression levels represents an important feature of tumor cells, resulting into an aberrant epigenetic regulation. Regarding liver tumor progression, miRNAs act as tumor suppressors (miR-122, miR26, and miR-223) or as oncogenic miRNAs (miR-130b, miR-221, and miR-222).

Emerging evidences suggest that miRNAs play a key role also in the maintenance, progression, chemoresistance, and disease relapse of HCSCs [4, 5]. For these reasons, many authors have identified in some mechanisms of “loss of stemness,” regulated by miRNAs expression, novel therapeutic strategies for treatment of hepatocellular carcinoma [91].

Here, we summarized the latest findings on the therapeutic targets of miRNA in HCSCs.

### 4.1. Oncogenic miRNA in HCSCs

Recently it has been demonstrated that miR-10b represents a switch factor between liver normal stem cells (LNSCs) and liver cancer stem cells (LCSCs). This malignant transformation is mediated by the enhanced expression of the axis miR-10b/HOX transcript antisense RNA (HOTAIR) that induces the degradation of E-cadherin pattern in LNSCs, thus facilitating the epithelial-to-mesenchymal transition (EMT) [34]. In this way also miR-21, when silenced, induces an attenuate mRNA expression of PTEN, RECK, or PDCD4, leading to a reduction in HCSCs migration and invasion [35]. The factors regulated by miR-21 represent also a target of miR-216a and miR-217 that are able to bind specifically PTEN and SMAD7. This leads to the activation of TGF-*β*1/PI3K/AKT signaling and to development of drug resistance to Sorafenib in HCC [53]. It has been showed that also miR-142-3p acts as an oncogenic via CD 133, conferring HCSC-like characteristic [39].

Several studies demonstrated that miR-155 acts as oncogenic miRNA, through the interaction with the axis TGF-*β*1/TP53INP1. This causes the EMT and the acquisition of stem cell phenotype [46, 92].

### 4.2. miRNA Tumor Suppressors in HCSCs

It is of note that miRNAs are able to regulate several biological mechanisms. For example, miR-122 has a key role in glycolytic metabolism. It induces a reversion of malignancy phenotype of HCSCs, by regulation of glycolysis, which is more active in HCSC CD133^+^, via inhibition of PDK4 and LDHA [37].

Several studies showed that the oncosuppressor miR-125b reduces EMT, through SMAD2/4 protein association [38]. This pathway seems to be influenced by the action of miR-148a, whose expression levels are improved by Glabridin (GLA) in HepG2, Huh-7, and MHCC97H hepatic cancer cell lines. MiR-125b inhibits TGF-*β*/SMAD2 axis and leads to lack of HCSC-like properties [41]. The isoform miR-148b instead acts on Neuropilin 1 (NRP1) with same effects [42]. In many studies, the interaction between some miRNAs and transcriptional factors has been described, such as Sox2, Oct4, Nanog, and c-myb, which play an important role in stemness maintenance [40, 44]. MiR-145, for example, plays a critical role in HCSCs tumor suppression, by reversing the effects of OCT-4 overexpression that normally leads to gain of tumorigenicity [40]. Conversely, miR-150 interacts with 3′UTR mRNA sequence of c-myb, downregulating its expression levels; in this case, it works as an oncosuppressor. Its presence is associated with a regression of HCSCs potential, probably due to a decrement of cyclin D1 and Bcl-2 levels [44].

A clinical study reported the dualistic effect of miR-150, also as an oncogene, together with miR-155 and miR-223. Their suppression is due to a decrement of EpCAM^+^ cell population [43].

Moreover, studies performed on miR-200a demonstrated that it regulates stemness of HCSCs with a dual activity. Overexpression of this miRNA switches on the transition from LCSC to HCSC that it is observed through the expression analysis of N-cadherin, ZEB2, and vimentin [50]. Another study on regulatory role of miR-200a showed that it acts as oncosuppressor in hepatic oval cells (HOCs), by direct interaction with Wnt/*β*-catenin axis. Functionally attenuation of miR-200a leads to the activation of the pathway, resulting in tumorigenicity acquisition after HOCs transition [49].

*β*-catenin represents also the molecular target of miR-214, which normally binds to the zeste homolog 2 (EZH2) factor by increasing EpCAM^+^ cells in HCC population. The attenuation of miR-214 or EZH2 overexpression leads to same results [52].

MiR-612 regulates the EMT through a direct interaction with AKT2 [56].

Recent studies have highlighted the key role of miR-181 in HCSCs stemness maintenance through the interaction with let-7 family members. It has been demonstrated that let-7/miR-181 axis is upregulated in HCSCs, and this condition leads to chemoresistance to doxorubicin or sorafenib treatment [48]. MiR-181 binds to some hepatic transcriptional regulators of differentiation as CDX2 and GATA6 or nemo-like kinase (NLK). These interactions induce the pluripotent phenotype, observed through an increment of EpCAM^+^ alpha-fetoprotein^+^ HCSCs [47].

Finally, recent studies reported the involvement of several miRNAs in HCSCs regulation/maintenance, through interaction with molecular target poorly studied. MiR-152, for example, shows an oncosuppressor role by targeting KIT receptor [45]; miR-205 and miR-491, acting as oncosuppressors, interact, respectively, with PLC*β*1 [51] and the GIT-1/NF-*κ*B axis [55].

The regulatory functions of miRNAs targeted in HCSCs are summarized in Table 2.

## 5. Therapeutic Targets of miRNA-Based Technology for Treatment of HCC

In order to eradicate the HCSCs, several therapeutic approaches have been developed. Here we summarized the recent progress in HCSCs research related to HCC, trying to provide a possible perspective for treatment of chemotherapy-resistant HCC tumors.

### 5.1. Epigenetic Therapy

Epigenetic mechanisms, such as histone modification and DNA methylation, play several roles in cancer development and progression [93]. Several studies showed the efficacy of epigenetic agents as therapeutic approach in HCC [94]. Raggi and colleagues in experimental studies of epigenetic reprogramming showed that Zebularine, a DNA methyltransferase (DNMT) inhibitor, is able to influence CSC properties such as self-renewal and tumorigenicity in HCSCs [95]. SALL4, a transcriptor factor, is able to regulate stemness of EpCAM-positive hepatocellular carcinoma, thus representing a valuable biomarker and therapeutic target for the diagnosis and treatment of HCC with stem cell features [96].

Altogether, these data suggest that epigenetic therapy may represent a promising approach for the eradication of CSC in HCC.

### 5.2. Antibody Therapy

Several studies suggested that targeting CSCs with monoclonal antibody could represent a strategy to improve the outcome of cancer therapy [97]. Regarding HCC, it has been proved that monoclonal antibodies have efficacy especially against CD13, EpCAM, and CD133, to eradicate the HCSCs [27, 98, 99]. Clinical trials and preclinical experiments will be necessary to confirm the safety of antibody therapy.

### 5.3. Molecular-Target Therapy

Molecular-target therapy is considered a promising therapeutic approach for HCC treatment. It has been demonstrated that self-renewal of colorectal CSC function is dependent on the BMI1 [100]. Other studies demonstrated that disruption of EZH2 impairs the tumor initiating, the self-renewal, and the cancer stem cells maintenance of various type tumors, including HCC [101–103]. Clinical trials would be needed to confirm if molecular-target therapy could be applied at clinical level, for HCSCs elimination.

### 5.4. Therapy Targeting the HCSCs Niche

Another type of therapy developed for the eradication of HCSCs is based on targeting the HCSCs niches. Niches are identified, as specific microenvironments in which HCSCs and normal tissue stem cells are present. It has been demonstrated that sorafenib, the unique molecular-target drug approved to treat HCC at clinical level, may contribute to the eradication of HCSCs by targeting Raf/MEK/ERK pathway and receptor tyrosine kinases [104, 105]. Clinical trials and preclinical experiments will be needed to confirm if therapy targeting the HCSCs niche could be considered innovative for HCC treatment.

### 5.5. miRNA in HCC Treatment

Recent evidences have suggested the potential application of miRNAs as novel strategy in cancer therapy for HCC. It has been previously described that the therapeutic application of miRNAs involves two different strategies [106, 107]. The first one inhibits oncogenic miRNAs by miRNA antagonists [108]. The second one is represented by miRNA replacement and is based on the reintroduction of a tumor suppressor miRNA mimetic to restore a loss of function [109]. One interesting study was performed in a mouse model of HCC by using mir-26a using adenoassociated virus delivered systemically. The authors demonstrated that ectopic expression of miR-26a leads to induction of tumor-specific apoptosis and to inhibition of cancer cell proliferation [110], indicating that delivery of miRNAs may provide an important therapeutic strategy in HCC treatment. However, its value in clinical trials still needs to be confirmed. To date, very few trials investigating the role of cancer-targeted miRNA in HCC have been performed. For example, a phase I trial investigating the role of drug MRX34, a liposome-based miR-34 mimic, is currently undergoing [111]. In order to assess the role of miRNA based drugs in clinical practice and in HCC treatment, more trials are necessary [112].

## 6. Conclusions

In this review, we synthesize the latest findings on therapeutic regulation of miRNA by modulation of tumor-suppressive and oncogenic signaling pathways. Data emerging from these studies suggest that deregulation of miRNA expression controls liver cancer progression and is responsible for the chemoresistance and disease relapse of HCSCs, although the underlying mechanisms are not completely elucidated. In order to highlight the perspective of novel miRNA-based anticancer therapies for HCC treatment, more studies will be needed in the future.

## Figures and Tables

**Table 1:** Cell surface marker of hepatic cancer stem cells.

Marker	Localization	Structure	Role in cancer	Ref.
CD133 (prominin 1)	Adult stem cells	Pentaspan transmembrane glycoprotein	Self-renewal, tumorigenicity, chemoresistance, and invasiveness	[23, 24]
CD90 (Thy-1)	Adult stem cells	Glycosyl-PI-anchored glycoprotein	Tumor formation, self-renewal, and metastasis	[25]
CD44	Mammalian cell types	Cell surface-glycoprotein	Tumor formation, chemoresistance, and metastatic ability	[26]
CD13	Predominantly on cells in G_1_/G_0_ phase	Zn^2+^ dep. Membrane bound ectopeptidase	Tumorigenicity, cell proliferation, self-renewal, and chemoresistance	[27]
CD24 (HAS)	Differentiating cells	Cell adhesion glycoprotein	Self-renewal, tumor formation, metastasis, and chemoresistance	[28]
OV6	Oval cells in fetal liver	Surface antigen	Tumor formation and chemoresistance	[29]
DLK1	Stem/progenitor hepatic and fetal liver cells	—	Proliferation, self-renewal, tumor formation, and tumor growth	[30]
EpCAM	Progenitors and stem cells	Transmembrane glycoprotein	Invasiveness, self-renewal, and tumor formation	[31]
GEP	Fetal liver	Hepatic oncofetal protein	Tumorigenicity and chemoresistance	[32]
SP (side population)	Cell subpopulation efflux chemotherapy drugs through ABC transporters	—	Tumorigenicity, self-renewal, and chemoresistance	[33]

**Table 2:** The regulatory functions of miRNAs in HCSCs biology.

miRNA	Oncogene (OG) oncosuppressor (OS)	Molecular target/pathways	Effects	Ref.
miR-10b	OG	HOX transcript antisense RNA (HOTAIR)	E-cadherin degradation EMT	[34]
miR-21	OG	PTEN, RECK, and PDCD4	Migration/invasion	[35]
miR-122	OS	PDK4, LDHA, and CD133	Glycolysis inhibition increased CD133^+^	[36, 37]
miR-125b	OS	SMAD2, SMAD4	EMT	[38]
miR-142-3p	OG	CD-133	HCSC features	[39]
miR-145	OS	OCT4	Tumorigenesis enhancing	[40]
miR-148a	OS	TGF-*β*/SMAD2	HCSC-features	[41]
miR-148b	OS	NRP1	HCSC-features	[42]
miR-150	OG/OS	3′UTR of mRNA c-myb	Cyclin D1/Bcl-2	[43, 44]
miR-152	OS	KIT	HCSC-features	[45]
miR-155	OG	TGF-*β*1/TP53INP1	EMT Decreased EpCAM^+^	[43, 46]
miR-181	OG	let-7 CDX2, GATA6, and NLK	Doxorubicin sorafenib resistance	[47, 48]
miR-200a	OG/OS	VASH2 in ZEB1/2 signaling Wnt/*β*-catenin	Transition LCSC/HCSC N-cadherin, ZEB2, and vimentin	[49, 50]
miR-205	OS	PLC*β*1	HCSC-features	[51]
miR-214	OS	EZH2 in Wnt/*β*-catenin signaling	EpCAM^+^	[52]
miR-216a	OG	PTEN and SMAD7 TGF-*β*1/PI3K/AKT	EMT Sorafenib resistance	[53]
miR-217	OG	PTEN and SMAD7 TGF-*β*1/PI3K/AKT	EMT Sorafenib resistance	[53]
miR-223	OG	—	HCSC-features	[43, 54]
miR-491	OS	GIT-1/NF-*κ*B	HCSC-features	[55]
miR-612	OS	AKT2	EMT	[56]
